# Development of 3D method to assess intramuscular spatial distribution of fat infiltration in patients with rotator cuff tear: reliability and concurrent validity

**DOI:** 10.1186/s12891-019-2631-z

**Published:** 2019-06-21

**Authors:** Rajan Khanna, Matthew D. Saltzman, James M. Elliott, Mark A. Hoggarth, Guido M. Marra, Imran Omar, Todd Parrish, Amee L. Seitz

**Affiliations:** 1 Stritch School of Medicine, Loyola University, Chicago, IL USA; 2 Department of Orthopedic Surgery, Feinberg School of Medicine, Northwestern University, Chicago, IL USA; 3 Northern Sydney Local Health District, The Kolling Research Institute & Faculty of Health Sciences, University of Sydney, Sydney, Australia; 4 Department of Physical Therapy & Human Movement Sciences, Feinberg School of Medicine, Northwestern University, Chicago, IL USA; 5 Department of Radiology, Feinberg School of Medicine, Northwestern University, Chicago, IL USA

**Keywords:** Dixon MRI, shoulder, muscle degeneration, Atrophy, Supraspinatus

## Abstract

**Background:**

Intramuscular fat infiltration is a critical factor in surgical decision-making and is the most important factor used to prognosticate surgical repair outcomes in patients with rotator cuff tears. Quantitative 3D assessment of total rotator cuff fat infiltration in patients with rotator cuff tears has been realized. However, a reproducible method to evaluate 3D spatial distribution of rotator cuff intramuscular fat has not been established. The objective of this study was to establish the reproducibility, change detectable beyond error, and concurrent validity of a semi-automated method to evaluate the 3D spatial distribution of fat infiltration and muscle volume in patients with rotator cuff tears.

**Methods:**

Thirteen consecutive patients diagnosed with symptomatic rotator cuff pathology and 3.0 T MRI confirmation at a single center were included. Fat-water imaging was used to quantify 3D intramuscular fat (%fat) in sagittal oblique sequences and intramuscular spatial distribution with the semi-automated technique. Each rotator cuff muscle was manually segmented yielding %fat in four axial intramuscular quartile-regions (superior-inferior; Q1–4) and three sagittal (medial/ intermediate/ lateral) regions. Reliability and concurrent validity of %fat and whole muscle volume were calculated with intraclass correlation coefficients (ICC).

**Results:**

Intra-rater reliability for intramuscular sagittal divisions (ICC = 0.93–0.99) and axial divisions (ICC = 0.78–0.99) was good/excellent. Inter-rater reliability for %fat (ICC = 0.82–0.99) and volume (ICC = 0.92–0.99) was good/excellent. Concurrent validity with commercialized software showed good/excellent agreement (ICC = 0.66–0.99).

**Conclusions:**

A new semi-automated method to assess 3-dimensional intramuscular distribution of fat infiltration in patients with rotator cuff tears using advanced MR imaging demonstrates high intra and inter-rater reliability and good concurrent validity. Minimal detectable change thresholds established facilitate clinical interpretation for future clinical application of this technique to assess change and treatment efficacy in patients with rotator cuff tears.

## Background

The fat infiltration and atrophy of the rotator cuff muscles are negative predictors of surgical outcomes [1–3] and, therefore, are critical factors to evaluate in the clinical management of patients with a symptomatic rotator cuff (RC) tear. Timely surgical repair of RC tear prior to progressive [4] and potentially irreversible physiological changes in muscle physiology [3] has been advocated, but non-surgical management in the cases of degenerative RC tear is also an effective option. Thus, the ability for clinicians to precisely detect the onset and rate of temporal rotator cuff muscle degeneration may assist with clinical decision making to determine the optimal treatment, timing, and efficacy of surgical repair for the individual patient with RC tear.

**Fig. 1:**
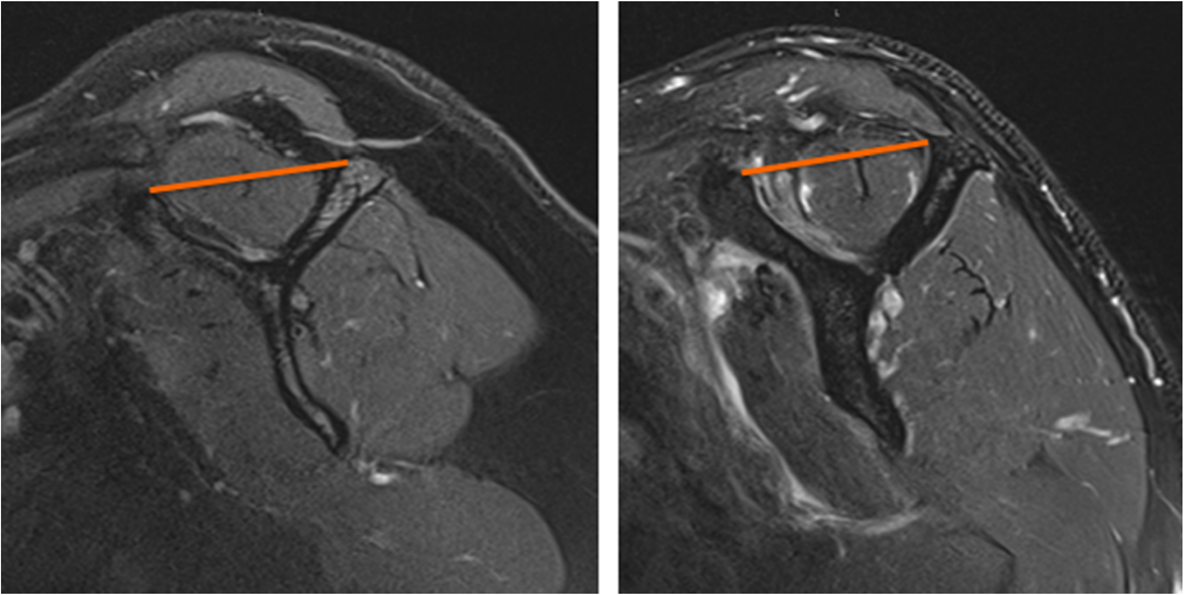
The Y-view used to clinically grade rotator cuff muscle fat infiltration and atrophy. Normal supraspinatus muscle (Left) and degeneration of the supraspinatus with atrophy and fat infiltration (Right). a tangent line (Orange) is used to evaluate the amount of atrophy

Muscle atrophy and fat infiltration are clinically evaluated in a sagittal oblique magnetic resonance image in a few slices at the Y-view (Fig. 1.). Atrophy is assessed clinically with an occupation ratio [5] or tangent line [6] permitting surgeons to visually estimate the muscle size relative to the surrounding bone (scapula). Fat infiltration of the rotator cuff muscles is also visually appreciated and rated in the clinic with clinic-friendly qualitative scales (i.e. Goutallier or Fuchs) [7, 8]. Despite the reported predictive value for these evaluative radiological scales to evaluate intramuscular fat, reproducibility and accuracy have been challenged [9, 10]. Additionally, the precision of these clinical scales do not allow for assessment of temporal changes that occur before significant moderate to severe degeneration negatively impacts the reparability and patient outcome [1–3]. For these reasons, more quantitative methods are necessary.

Advanced MRI techniques that allow for greater precision and accuracy towards quantifying intramuscular fat by using chemical shift–based water-fat techniques, such as the Dixon [11] or IDEAL methods are more readily available. With these methods, data are collected at an echo time when water and fat are in-phase and at an echo time when the phases are opposed. The data can then be combined to generate a fat and water image allowing for calculation of percent fat infiltration (%fat). These quantitative techniques are not new and have been used to evaluate the magnitude of muscle %fat in individuals with a variety of musculoskeletal disorders [12–15] and have demonstrated concurrent validity with positive correlation to clinical grading of fat, [16] tear size, [17] and biopsy results [18].

Typically quantitative techniques have assessed %fat in a single slice or several slices near the scapular y-view have demonstrated construct validity with associations to the clinical Goutallier grading of muscle [16] and the size of the tear [17]. However, the accuracy of a 2D assessment in a single or few y-view slice images has also been challenged in the presence of tendon retraction or with inhomogeneous degenerative changes. Recently, Matsumura, et al. [19] established the reliability of 3D assessment for intramuscular fat% taking the average of all slices across the entire muscle in patients with rotator cuff tears. While such work clearly advances our understanding of total muscle composition, the technique does not capture or appreciate the heterogeneity of intramuscular fat infiltration in rotator cuff tears [20]. The ability to appreciate inhomogeneous distribution of intramuscular fat within the rotator cuff muscles will facilitate further understanding of the temporal progression of muscle degeneration with rotator cuff tears. Yet, there are currently no methods to evaluate spatial distribution of intramuscular %fat of the rotator cuff with established reliability or validity.

Thus, we seek to expand on the existing 3D imaging methods used to quantify physiological degenerative changes in rotator cuff muscles by first, establishing measurement properties using sagittal oblique slices, and second to report on the feasibility and reliability of a novel semi-automated method to assess the 3D intramuscular fat distribution to allow for detection of inhomogeneous changes. The purpose of this study is to establish the reproducibility, change detectable beyond measurement error (MDC), and concurrent validity to define 3D whole muscle volume and spatial distribution of intramuscular fat% in both the axial and sagittal planes in patients with rotator cuff tears.

## Methods

### Subjects

This study was a single-center study of patients seen by one of two fellowship trained shoulder surgeons between December 2015 and May 2016. Consecutive patients diagnosed with rotator cuff tendon pathology and confirmed with MR imaging at this institution were retrospectively identified and included in the study. This subset of patients was selected to maintain consistency in the scanner used for MR imaging and sequences available for analysis. The surgeon diagnosis of rotator cuff tendon pathology included a positive finding in at least 3 of the following clinical tests: Hawkins Test, Neer Sign, Painful Arc, External Rotation Lag Sign, Hornblower Sign, Pain with External Rotation Resistance or a Jobe Sign. Exclusion criteria included a limitation in passive shoulder range of motion in 3 or more planes consistent with adhesive capsulitis. Patients with moderate to severe glenohumeral joint osteoarthritis, prior rotator cuff repair or other shoulder surgery, and patients with history of a shoulder fracture or deformity from prior fractures were also excluded. A fellowship trained, board certified musculoskeletal radiologist with over 12 years of experience, independently read the MR images blinded to the surgeon diagnosis. Patients determined to have both symptomatic clinical diagnosis and findings on MRI of rotator cuff pathology were included. Rotator cuff tendon pathology was classified by the radiologist as no tear, partial thickness tear, and full-thickness tear small < 1 cm, medium 1–3 cm, large > 3–5 cm, or massive > 5 cm. The radiologist also qualitatively graded fat infiltration using the Goutallier Classification. Using standardized methods, [21] the sample size estimate was 10 subjects using a one-sided α = 0.05, and β = 0.80 to yield 80% power to detect an acceptable reliability Intraclass Correlation Coefficient (ICC) of 0.75 and an expected ICC of 0.95 based on previous research [19]. We, therefore, included the first 13 patients meeting inclusion/exclusion criteria in this study. The study was approved by the Institutional Review Board and conducted in accordance with the Committee for Human Research.

### Shoulder MRI protocol

Magnetic resonance images were acquired according to service institutional standards using a 3-T Siemens (Skyra, Siemens, Erlangen, Germany) magnetic resonance scanner with a 16-channel phased array shoulder coil. To ensure coverage of the rotator cuff muscles, the sagittal oblique MR imaging sequence with respect to the glenoid fossa was planned on an axial scout scan with a field of view of 18 × 18 cm, which was fit to include the medial border of the scapula. T1-weighted sequences which include fast spin-echo sequences in oblique coronal and sagittal planes, a multiple echo data image continuation (MEDIC) sequence in the axial plane and a T2*-weighted fat suppressed sequences in the axial plane, coronal oblique, and sagittal oblique planes were also performed. Finally, a 3D multi-echo two-point Dixon fat/water imaging sequence was performed in the sagittal oblique plane. This sequence is a chemical-shift imaging application producing water- and fat-only images from dual echo acquisitions with precise accuracy for %fat quantification [18, 22, 23]. The imaging parameters were as follows: slice thickness 2.0 mm, TR/TE1/TE2 = 3.97 ms/1.29 ms/2.52 ms, Flip angle 9°, 380 mm FOV, and acquisition matrix of 320 × 320 with 120 slices to produce a voxel resolution of 1.2 × 1.2 × 2.0 mm and 1040 Hz/Px bandwidth. In order to reduce aliasing in both the phase and 3D direction, oversampling of 100 and 60% were used respectively. To reduce the acquisition time, an acceleration factor of 2 was used in both the slice and phase directions.

### Image analysis

The 3D intramuscular %fat and muscle volume were quantified by manual segmentation of rotator cuff muscles on the Dixon fat-water sequences using custom software in Matlab (Mathworks V, Natick MA). Two examiners without experience reading MR images were trained in two 30-min sessions by a musculoskeletal radiologist and 2 orthopedic surgeons to identify the boarders of the regions of interest. To establish inter-rater reliability, both examiners independently performed segmentation of the regions of interest on fat-water sequence MR images, blinded to subject identifiers, tear size, and each other’s results. To establish feasibility and intra-rater test-retest reliability, one examiner repeated the segmentation 6-weeks later also blind to subject identifiers; tear size, and prior segmentation results. Lastly, we examined concurrent validity of %fat and muscle volumes with manual segmentation of the regions of interest using commercially available software (AnalyzeDirect Software, V. 11.0) with the same fat-water imaging processing parameters. This system has been used to assess intramuscular fat [12–15] and previously validated with the gold-standard, biopsy [18].

**Fig. 2:**
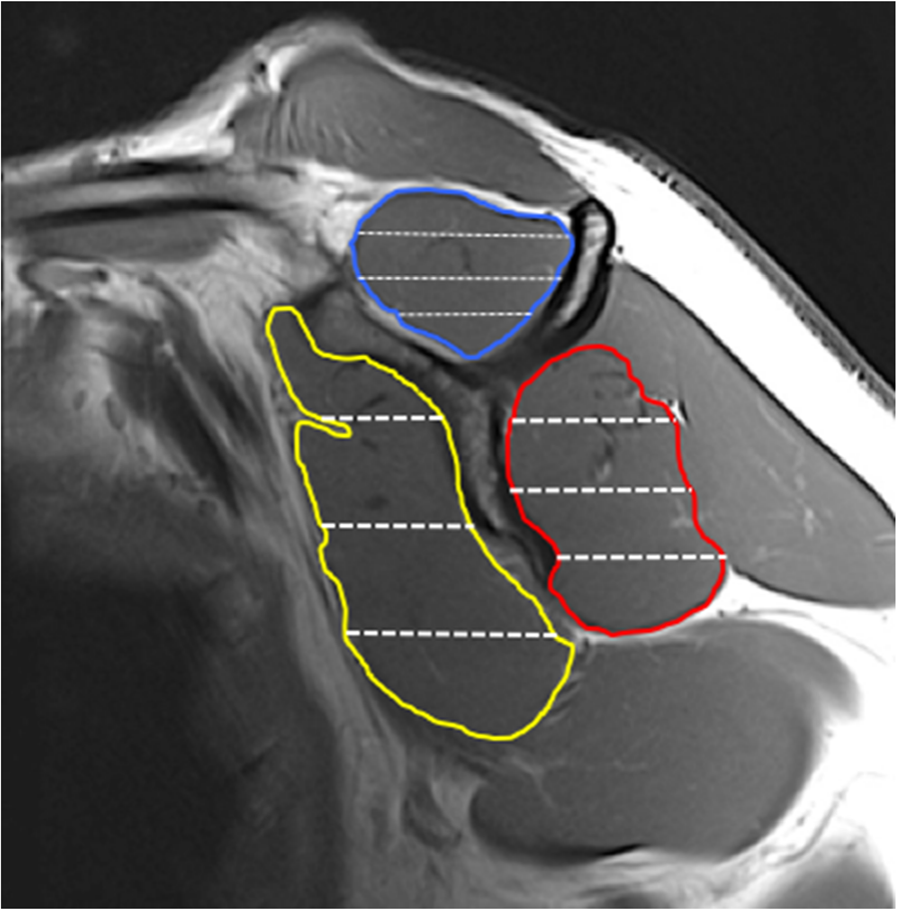
Manual segmentation of the supraspinatus (Blue), combined infraspinatus/teres minor (Red), subscapularis (Yellow). Spatial distribution of intramuscular %fat in automated superior to inferior quartiles was derived for each muscle

All segmentation was performed manually inside the fascial borders the defined the regions of interest in supraspinatus (SS), combined infraspinatus/teres minor (IS), subscapularis (SC) muscles in each oblique sagittal image (Fig. 2.). The MATLAB program generated %fat data for quartilesin each of the segmented muscles to evaluate spatial distribution of %fat by quartile superiorly to inferiorly (Q1-Q4) (Fig. 2.). Consistent with methods described by prior investigators, the infraspinatus and teres minor muscles were segmented and evaluated as one [6, 24, 25]. Using these muscular regions of interest, two sets of co-registered fat-water images were used to calculate fat fraction (%fat) with the following equation:

Fat Fraction = Fat/(Fat + Water) × 100.

**Fig. 3:**
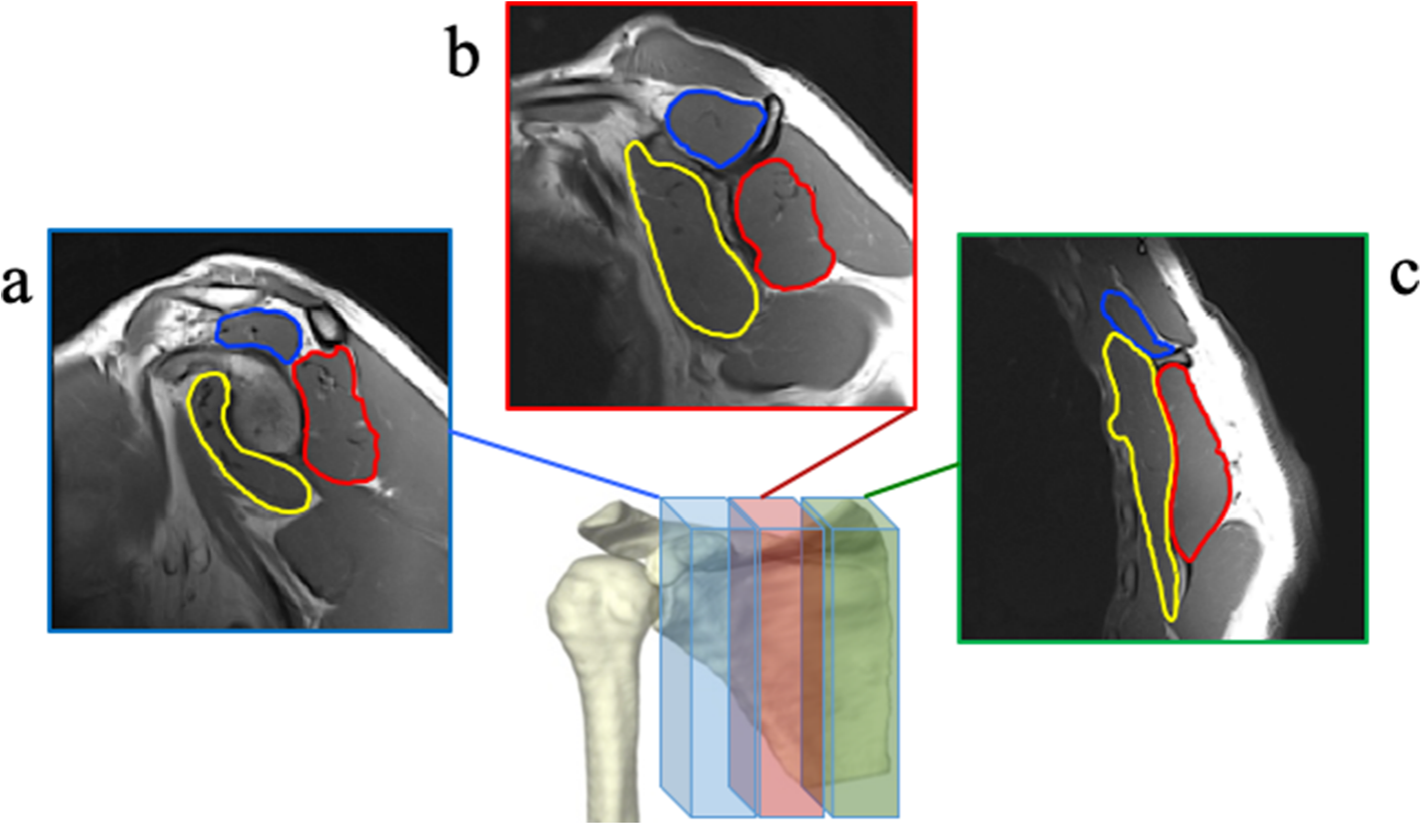
Reliability of the spatial distribution of %fat was calculated to derive %fat in the: **a**) lateral, **b**) intermediate and **c**) medial intramuscular tertile for each rotator cuff muscle. The mean total muscle %fat and volume were calculated from all regions

Sequential images from the most lateral image at the level of the glenoid to the most medial image of the medial scapular boarder were processed. Thus, the number of images used in the oblique sagittal view varied by subject, due to individual subject size differences needed to capture the scapula and corresponding rotator cuff muscles. The average of all consecutive images was used to establish the 3D total muscle %fat of each muscle. The 3D volume of each muscle was calculated by using the sum of the area calculated in each slice taking into account slice thickness from the glenoid to the medial boarder of the scapula. In addition to 3D whole muscle measures, three sagittal intra-muscular tertiles (lateral, intermediate, and medial) were created by dividing each muscle from the glenoid to the medial boarder of the scapula into equal **thirds (**Fig. 3**)** whereby average %fat and volume of each intra-muscular region were calculated.

### Statistical analysis

The relative intra and inter-rater reliability of 3D %fat and volume of the entire muscle and intramuscular divisions were determined by calculating the Intraclass Correlation Coefficient (ICC) [26] and 95% Confidence Intervals (95%CI). For all analyses, the ICC values were considered: poor if below 0.20; fair from 0.21 to 0.40; moderate from 0.41 to 0.60; good from 0.61 to 0.80; and very good from 0.81 to 1.00 [27]. The absolute reliability was defined as the standard error of the measure (SEM), representing error associated with a single measure [28], and the minimal detectable change (MDC), representing the value that exceeds error associated with repeated measures [29]. The MDC is used to interpret changes in fat infiltration and volume that are necessary to exceed measurement error. The concurrent validity was also determined with ICCs by comparing values between those derived with current methods and previously validated commercial software. All statistical analyses were conducted with the IBM SPSS Statistics software (Version 23; IBM Corp, Armonk, NY).

## Results

Intra and inter-rater reliability was assessed in MR imaging of 13 consecutive patients (5 males, 8 females) who met the inclusion criteria. These patients had a mean age of 51.0 ± 16.5 (±SD) years with 7 patients with partial-thickness tears, 2 patients with small full-thickness tears, and 4 patients with medium -sized full-thickness tears. Goutallier grade 2 fat infiltration was identified in the supraspinatus in one patient, and grade 1 fat infiltration in 4 patients. The infraspinatus showed grade 1 fat infiltration in 4 patients, and the teres minor showed grade 1 fat infiltration in one patient. The mean chronicity of patient’s symptoms was 38.5 ± 78.0 months. The intra-rater absolute reliability of spatial distribution methods evaluating 3D fat infiltration and 3D muscle volume of the rotator cuff muscles was considered very good to excellent from medial to lateral tertiles (Table 1) and %fat (Table 2) from superior to inferior quartiles (Q1-Q4). Relative reliability, with the SEM and MDC expressed in units of %fat, volume was also established and shown. The inter-rater absolute reliability assessing the spatial distribution of 3D fat infiltration and volume of the rotator cuff muscles was considered good to excellent from medial to lateral tertiles (Table 3) and %fat (Table 4) from superior to inferior quartiles (Q1-Q4). With regard to current validity (Table 5), the ICCs for agreement between the two methods was also very good to excellent for both fat% and volume across the entire muscle.

**Table 1:** Intra-rater reliability of 3D %fat in superior to inferior quartile (Q1-Q4) regions in patients (*N* = 13) with rotator cuff pathology

Intra-rater Reliability					
	3D %Fat				
Rotator Cuff Muscle	Absolute		Relative		
Intramuscular region	ICC	95% CI	Mean	SEM	MDC
Supraspinatus (whole)					
Q1	0.960	0.872, 0.988	13.85	1.74	2.46
Q2	0.991	0.971, 0.997	12.85	0.73	1.04
Q3	0.988	0.961, 0.996	12.43	0.85	1.20
Q4	0.927	0.770, 0.977	14.19	2.38	3.36
Infra/Teres (whole)					
Q1	0.993	0.977, 0.998	10.38	0.55	0.78
Q2	0.994	0.981, 0.998	11.42	0.61	0.86
Q3	0.984	0.948, 0.995	12.50	0.96	1.36
Q4	0.945	0.821, 0.983	10.96	1.54	2.18
Subscapularis (whole)					
Q1	0.782	0.280, 0.934	13.04	2.95	4.17
Q2	0.996	0.987, 0.999	13.77	0.54	0.77
Q3	0.998	0.994, 0.999	13.77	0.44	0.64
Q4	0.995	0.963, 0.999	15.35	0.75	1.06

*ICC* intraclass correlation coefficient, 95% CI, 95% confidence interval, *SEM* standard error of the measure, *MDC* minimal detectable change

**Table 2:** Intra-rater reliability of 3D %fat and volume in medial to lateral (tertile) regions patients (*N* = 13) with rotator cuff pathology

	Intra-rater Reliability									
	3D %Fat					3D Volume (cm3)				
Rotator Cuff Muscle	Absolute		Relative			Absolute		Relative		
Intramuscular region	ICC	95% CI	Mean	SEM	MDC	ICC	95% CI	Mean	SEM	MDC
Supraspinatus (whole)	0.974	0.915, 0.992	11.69	1.10	1.56	0.958	0.869, 0.987	29.6	2.9	4.1
Lateral Region	0.935	0.794, 0.980	10.38	1.20	1.70	0.689	−0.036, 0.906	8.1	1.9	2.7
Intermediate Region	0.980	0.936, 0.994	11.65	1.08	1.53	0.967	0.890, 0.990	11.4	1.0	1.4
Medial Region	0.975	0.918, 0.992	13.03	1.49	2.11	0.996	0.988, 0.999	10.1	0.4	0.6
Infra/Teres (whole)	0.989	0.966, 0.997	10.51	0.68	0.96	0.979	0.929, 0.994	65.7	3.5	5.0
Lateral Region	0.968	0.897, 0.990	10.54	1.12	1.58	0.935	0.789, 0.980	19.1	1.7	2.4
Intermediate Region	0.977	0.927, 0.993	10.76	1.00	1.42	0.976	0.924, 0.993	23.0	1.2	1.8
Medial Region	0.957	0.865, 0.987	10.22	1.41	1.99	0.978	0.920, 0.993	23.6	1.8	2.5
Subscapularis (whole)	0.996	0.986, 0.999	13.12	0.47	0.67	0.910	0.701, 0.973	78.2	8.1	11.4
Lateral Region	0.984	0.945, 0.995	13.73	0.97	1.38	0.879	0.595, 0.963	20.5	3.2	4.6
Intermediate Region	0.996	0.987, 0.999	13.14	0.48	0.68	0.983	0.945, 0.995	31.6	1.5	2.1
Medial Region	0.995	0.984, 0.999	13.04	0.56	0.79	0.956	0.797, 0.988	26.3	2.8	3.9

*ICC* intraclass correlation coefficient, 95% CI, 95% confidence interval, *SEM* standard error of the measure, *MDC* minimal detectable change

**Table 3:** Inter-tester reliability of 3D %fat and volume for whole muscle and medial to lateral (tertile) regions in patients (*N* = 13) with rotator cuff pathology

	Inter-rater Reliability									
	3D %Fat					3D Volume				
Rotator Cuff Muscle	Absolute		Relative (%fat)			Absolute		Relative (cm3)		
Intramuscular region	ICC	95% CI	Mean	SEM	MDC	ICC	95% CI	Mean	SEM	MDC
Supraspinatus (whole)	0.979	0.861, 0.995	11.03	0.87	1.22	0.971	0.910, 0.991	29.9	2.5	3.5
Lateral Region	0.820	0.218, 0.950	8.88	1.73	2.45	0.735	0.160, 0.918	7.9	1.9	2.7
Intermediate Region	0.984	0.930, 0.996	11.26	0.89	1.25	0.982	0.940, 0.994	11.8	0.7	1.0
Medial Region	0.948	0.829, 0.984	12.80	1.81	2.56	0.989	0.966, 0.997	10.1	0.7	0.9
Infra/Teres (whole)	0.992	0.947, 0.998	10.05	0.55	0.78	0.973	0.897, 0.992	69.3	4.2	6.0
Lateral Region	0.990	0.968, 0.997	10.50	0.65	0.91	0.923	0.745, 0.977	19.5	2.0	2.8
Intermediate Region	0.990	0.931, 0.998	10.19	0.64	0.90	0.966	0.824, 0.991	24.3	1.5	2.2
Medial Region	0.984	0.940, 0.995	9.53	0.76	1.07	0.944	0.820, 0.983	25.5	2.7	3.8
Subscapularis (whole)	0.996	0.989, 0.999	12.75	0.47	0.66	0.955	0.857, 0.986	83.1	6.2	8.8
Lateral Region	0.989	0.964, 0.997	13.34	0.79	1.12	0.629	0.369, 0.889	20.1	4.5	6.4
Intermediate Region	0.990	0.952, 0.997	12.60	0.77	1.09	0.962	0.874, 0.988	32.9	2.4	3.4
Medial Region	0.996	0.987, 0.999	12.20	0.51	0.72	0.840	0.473, 0.951	30.1	4.9	6.9

*ICC* intraclass correlation coefficient, 95% CI, 95% confidence interval, *SEM* standard error of the measure, *MDC* minimal detectable change

**Table 4:** Inter-rater reliability of 3D %fat in superior to inferior quartiles (Q1-Q4) in patients (*N* = 13) with rotator cuff pathology

	Inter-rater Reliability				
	3D %Fat				
Rotator Cuff Muscle	Absolute		Relative		
Intramuscular region	ICC	95% CI	Mean	SEM	MDC
Supraspinatus (whole)					
Q1	0.980	0.936, 0.994	13.26	1.09	1.54
Q2	0.978	0.879, 0.994	12.27	1.05	1.48
Q3	0.982	0.881, 0.995	12.12	0.98	1.39
Q4	0.926	0.300, 0.983	12.03	1.73	2.45
Infra/Teres (whole)					
Q1	0.996	0.986, 0.999	10.09	0.40	0.57
Q2	0.997	0.989, 0.999	11.18	0.42	0.59
Q3	0.984	0.946, 0.995	11.85	0.90	1.28
Q4	0.963	0.469, 0.992	9.87	1.13	1.60
Subscapularis (whole)					
Q1	0.991	0.971, 0.997	11.84	0.51	0.72
Q2	0.996	0.983, 0.999	13.25	0.54	0.76
Q3	0.996	0.986, 0.999	13.38	0.61	0.86
Q4	0.997	0.991, 0.999	14.89	0.58	0.82

*ICC* intraclass correlation coefficient, 95% CI 95% confidence interval, *SEM* standard error of the measure; MDC, minimal detectable change

**Table 5:** Concurrent validity of 3D total and medial to lateral region %fat and volume

	Validity			
Rotator Cuff Muscle	3D %Fat		Volume	
Intramuscular region	ICC	95% CI	ICC	95% CI
Supraspinatus (whole)	0.859	0.015, 0.970	0.939	0.276, 0.987
Lateral Region	0.655	0.250, 0.907	0.778	0.070, 0.942
Intermediate Region	0.844	0.083, 0.963	0.867	0.153, 0.968
Medial Region	0.947	0.830, 0.984	0.884	0.675, 0.963
Infra/Teres (whole)	0.989	0.965, 0.997	0.969	0.889, 0.991
Lateral Region	0.969	0.905, 0.991	0.882	0.384, 0.969
Intermediate Region	0.976	0.924, 0.993	0.937	0.780, 0.981
Medial Region	0.980	0.968, 0.997	0.911	0.743, 0.972
Subscapularis (whole)	0.975	0.872, 0.993	0.912	0.313, 0.979
Lateral Region	0.951	0.852, 0.985	0.580	0.037, 0.854
Intermediate Region	0.978	0.907, 0.994	0.927	0.192, 0.985
Medial Region	0.949	0.694, 0.987	0.885	0.615, 0.965

*ICC* intraclass correlation coefficient, 95% CI 95% confidence interval, *SEM* standard error of the measure, *MDC* minimal detectable change

## Discussion

Results of this study show the feasibility of advanced MR imaging techniques using a novel semi-automated method to quantify the spatial distribution of 3D rotator cuff muscle fat infiltration in patients with rotator cuff pathology. Furthermore, these methods demonstrate good to excellent reliability and concurrent validity with previously validated fat fraction and volumetric methods [18].

In prior studies using quantitative 2D methods, reliability of %fat with intraclass correlation coefficients (ICC) of 0.60 for the supraspinatus, 0.65 of the infraspinatus using 4 consecutive lateral slices adjacent to the sagittal oblique “scapular y-view” were reported [16]. Another study reported higher test-retest reliability of %fat in the rotator cuff ranging from ICC = 0.895 to 0.952 [30]. Similarly, volume estimates of the entire muscle have been performed using selected cross-section area measures of the muscle [31] and cross-sectional area based atrophy ratios [6]. However, recent evidence suggests there is variation in %fat regionally within the rotator cuff muscles in individuals with rotator cuff pathology [20]. Thus 2D measures using a single slice or several consecutive slices adjacent to the y-view may not provide the most sensitive test towards detecting the disparate spatial distribution and magnitude of temporal changes in fat infiltration.

To adequately evaluate temporal changes of fat and atrophy in rotator cuff muscles in patients with tendon pathology, 3D quantification could appreciate early inhomogeneous degeneration following a rotator cuff tear before irreversible change occurs. Matsumura et al., recently demonstrated excellent reliability with 3D %fat and volume using a standardized protocol (3 T, 1 mm slice) in axial slices [19]. Our results show comparable 3D whole muscle %fat and volume data. Furthermore, the technical advances in 3D methods in the current study provide feasibility and reliability of a semi-automated technique to quantify intramuscular distribution medial- lateral (tertiles) and superior-inferior (quartiles) to facilitate quantification of inhomogeneous 3D fat infiltration. Since muscles are 3D structures, imaging methods that best capture 3D rotator cuff muscle fat and atrophic changes of the in patients with rotator cuff tears [19]. Spatial distribution of %fat using the Dixon sequence in cervical and lumbar spine musculature has been established [13, 22, 32]. However, to our knowledge the reliability of evaluating the spatial distribution of %fat of rotator cuff muscles has not been previously reported, which supports the novelty of the processing methods. Additionally, the current study demonstrates excellent reliability and concurrent validity of 3D whole muscle and intramuscular regional distribution assessment of %fat and volume with commercial software, providing additional support of the validity of our methods.

In addition to establishing absolute reliability, we also present error threshold data relevant to interpreting meaningfulness of statistical results for fat infiltration and volume measures. The SEM and MDC are the errors associated with a single measurement and repeated measures, respectively, in the units of measure (%fat and cm^3^ volume). The intra-rater MDC was less than 2% for each rotator cuff muscle and less than 2.2% for the intramuscular spatial distribution measures of %fat within the lateral, intermediate, and medial regions of each muscle using our method (Table 1). However, the error of measurement established by the MDC in the superior-inferior quartiles were higher (Table 2) for all muscles (< 2.5%) with the exception of Q1- the most superior aspect of the subscapularis (MDC = 4.17%). The inter-rater MDC (Tables 3 and 4) was less than 1% for each rotator cuff muscle and intramuscular spatial distribution errors were all less than 2.6% in both the superior-inferior (quartiles) and medial to lateral (tertiles). This error threshold is important to assist interpretation of emerging research evaluating the efficacy of a healed surgical tendon repair to negate or potentially reverse rotator cuff muscle %fat in patients with rotator cuff tear [30, 33].

There are several limitations to the study. Overall, the generalizability may be limited as the results were obtained using a single scanner and set of imaging parameters. Additionally, we only examined the intra-rater reliability in one rater and both raters did not have MR image reading experience. While the study exceeded our sample size estimate to provide > 80% power, sample was small (*n* = 13). However, our results are consistent with the precision previously established, and the time intensive process of manually segmenting each muscle in its entirety limits further efforts for this purpose. Furthermore, we did not include patients with large and massive rotator cuff tears as early detection of temporal change in muscle degeneration is most imperative in patients with less severe rotator cuff disease, such as partial thickness and small-medium full-thickness tears. In these tears, operative treatment is advocated before potentially irreversible changes in the rotator cuff muscle occurs. Thus the population we studied were those who may potentially benefit the most from assessment of temporal physiological muscle changes pre-operatively. Additionally, we combined and segmented the infraspinatus and teres minor together based on previous literature [6] and the difficulty visualizing the fascia plane in sagittal Y-views. However, with the evolution of improved imaging technologies, it is anticipated that the reliable differentiation between these two muscles in sagittal oblique images will be realized and this work is currently underway. Lastly, the pattern of fat infiltration in patients with rotator cuff tears may provide further insight into early temporal changes in muscle degeneration in patients with rotator cuff tears.

## Conclusions

3D MR imaging techniques including, the multi-echo Dixon fat-water sequences allow for reliable and intra- and inter-rater quantification of the intramuscular spatial distribution of fat infiltration in patients with rotator cuff pathology. It is suggested that both atrophy and fat infiltration should be evaluated separately, given these are two different physiological processes [3, 34, 35]. To adequately evaluate temporal changes of fat and atrophy in rotator cuff muscles in patients with tendon pathology, 3D quantification may be necessary to determine the rate and distribution of muscle degeneration following a rotator cuff tear. While conservative treatment may be advocated for patients with a degenerative rotator cuff tear, determining the rate of muscle degeneration would aid in surgical repair timing decisions. Current study results suggest this method to quantify 3D spatial distribution of muscle pathophysiology is feasible and has utility to reliability quantify rotator cuff muscle degeneration. As future studies utilize more precise 3D measures of muscle atrophy and fat infiltration, the ability to determine the meaningfulness of whether statistical differences exceed measurement error is important; thus the absolute error in units of %fat was established. The clinical application of this method to evaluate the spatial distribution muscle degeneration may be useful to facilitate surgical and non-surgical treatment choices in patients with rotator cuff tears.

## Abbreviations

%fat: percent fat infiltration
3D: Three-dimensional
95%CI: 95% Confidence Interval
ICC: Intraclass Correlation Coefficient
MDC: minimal detectable change
RC: Rotator Cuff
SEM: Standard error of the measure
